# African swine fever: an unprecedented disaster and challenge to China

**DOI:** 10.1186/s40249-018-0495-3

**Published:** 2018-10-26

**Authors:** Tao Wang, Yuan Sun, Hua-Ji Qiu

**Affiliations:** State Key Laboratory of Veterinary Biotechnology, Harbin Veterinary Research Institute, Chinese Academy of Agricultural Sciences, Harbin, 150069 China

**Keywords:** African swine fever, Control, China

## Abstract

**Background:**

African swine fever (ASF), caused by African swine fever virus, is a hemorrhagic and often fatal disease of domestic pigs and wild boar, which is notifiable to the World Organization for Animal Health. On August 3, 2018, China reported the first outbreak of ASF in Shenyang, a northeastern city of China. As of October 8, a total of 33 ASF outbreaks were reported in eight provinces in China, the biggest pork producer and consumer in the world.

**Main body:**

This commentary summarizes the current situation of ASF in China, measures that China has taken to control the disease, lessons learnt from other countries, challenges and recommendations on ASF control in China, and possible international collaborations on ASF.

**Conclusions:**

ASF is an unprecedented disaster and challenge to the Chinese swine industry. It will be a formidable and protracted campaign to control ASF in China, which requires joint participation and coordination of stakeholders and agencies at different levels.

**Electronic supplementary material:**

The online version of this article (10.1186/s40249-018-0495-3) contains supplementary material, which is available to authorized users.

## Multilingual abstracts

Please see Additional file 1 for translations of the abstract into the five official working languages of the United Nations.

## Background

**Fig. 1:**
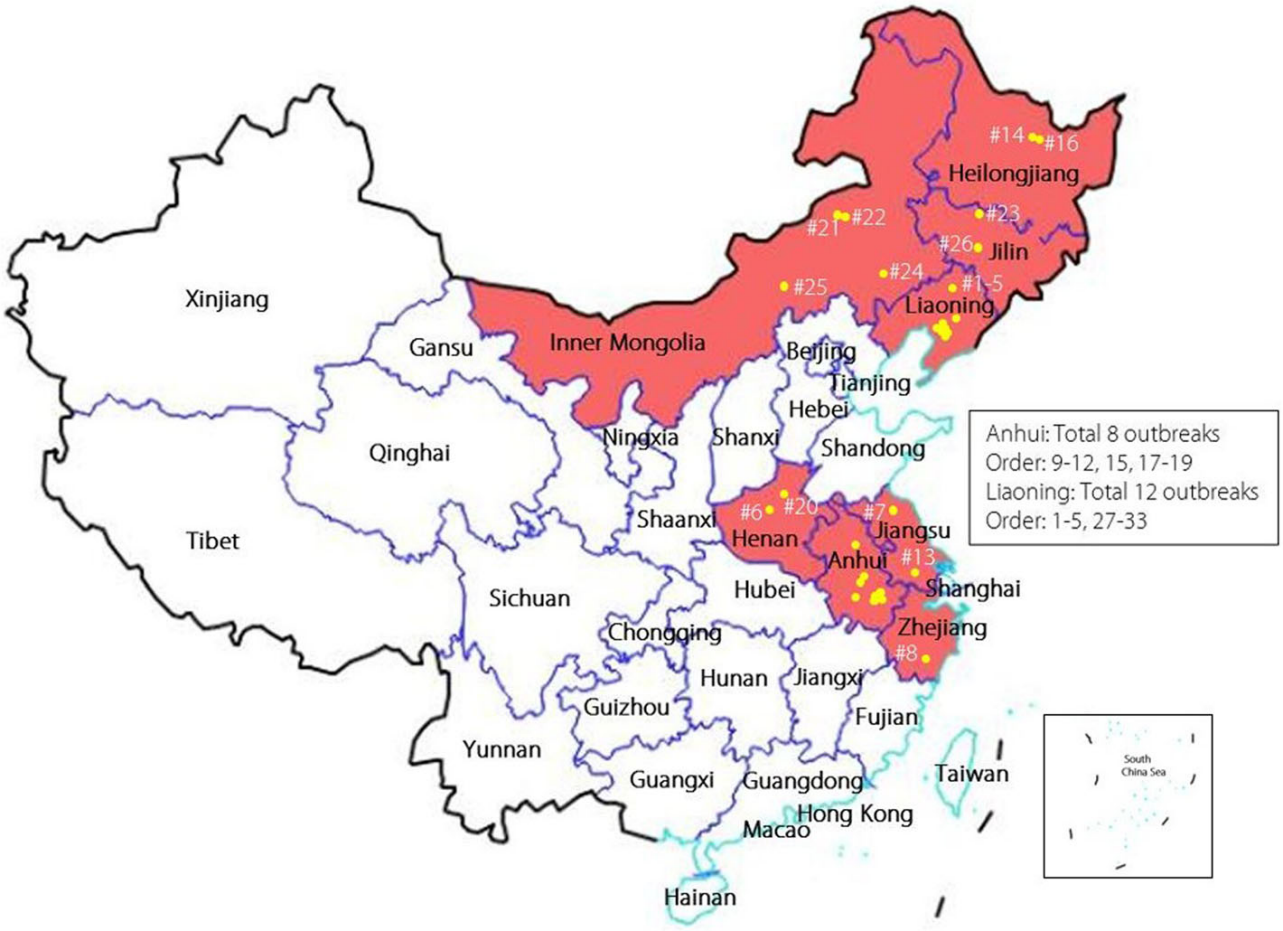
African swine fever outbreak locations in China. The provinces with African swine fever outbreaks are shaded in red; numbers and dots indicate the order and locations

On August 3, 2018, China reported an outbreak of African swine fever (ASF) in Shenyang, a northeastern city of China [1]. This is the first emergence of ASF in China, which is the largest pork producer and consumer in the world. As of October 8, a total of 33 ASF outbreaks have been reported in eight provinces in the country (Fig. 1; Table 1). Though the ASF viral sequence in China is highly homologous to that of the Georgia 2007/1 strain [2], it remains a mystery where and how the virus came from [3]. It is speculated that the disease might have been introduced from an ASF-affected Eastern European country via smuggled pork or offals.

**Table 1:** Summary of African swine fever outbreaks in China

Order	Date of confirmation	Locations (City, Province)	Susceptible	Cases	Deaths	Unit type
1	Aug. 3, 2018	Shenyang, Liaoning	19 420	47	47	Farm
2	Aug. 7, 2018	Shenyang, Liaoning	160	3	0	Farm
3	Aug. 7, 2018	Shenyang, Liaoning	55	1	0	Farm
4	Aug. 7, 2018	Shenyang, Liaoning	216	2	0	Farm
5	Aug.7, 2018	Shenyang, Liaoning	140	1	0	Farm
6	Aug. 16, 2018	Zhengzhou, Henan	1806	30	30	Slaughterhouse
7	Aug. 19, 2018	Lianyungang, Jiangsu	14 686	615	88	Farm
8	Aug. 22, 2018	Wenzhou, Zhejiang	1864	430	340	Farm
9	Aug. 30, 2018	Wuhu, Anhui	459	185	80	Farm
10	Sept. 2, 2018	Xuancheng, Anhui	285	63	42	Farm
11	Sept. 2, 2018	Xuancheng, Anhui	440	153	111	Farm
12	Sept. 3, 2018	Xuancheng, Anhui	308	152	83	Farm
13	Sept. 3, 2018	Wuxi, Jiangsu	97	12	9	Farm
14	Sept. 5, 2018	Jiamusi, Heilongjiang	87	39	12	Backyard
15	Sept. 6, 2018	Chuzhou, Anhui	886	62	22	Farm
16	Sept. 6, 2018	Jiamusi, Heilongjiang	203	26	10	Farm
17	Sept. 6, 2018	Wuhu, Anhui	30	13	4	Farm
18	Sept. 6, 2018	Xuancheng, Anhui	52	15	15	Farm
19	Sept. 10, 2018	Tongling, Anhui	219	63	23	Farm
20	Sept. 14, 2018	Xinxiang, Henan	2087	148	64	Farm
21	Sept. 14, 2018	XilinGol League, Inner Mongolia	237	16	16	Backyard
22	Sept. 17, 2018	XilinGol League, Inner Mongolia	159	14	8	Backyard
23	Sept. 21, 2018	Gongzhuling, Jilin	484	56	56	Farm
24	Sept. 21, 2018	Hinggan League, Inner Mongolia	138	23	22	Backyard
25	Sept. 24, 2018	Hohhot, Inner Mongolia	388	4	2	Slaughterhouse
26	Sept. 28, 2018	Songyuan, Jilin	44	8	3	Farm
27	Sept. 30, 2018	Yingkou, Liaoning	130	22	22	Village
28	Sept. 30, 2018	Yingkou, Liaoning	9	2	2	Village
29	Sept. 30, 2018	Yingkou, Liaoning	239	78	78	Village
30	Oct. 7, 2018	Yingkou, Liaoning	1337	65	20	Village
31	Oct. 7, 2018	Yingkou, Liaoning	2608	127	27	Village
32	Oct. 7, 2018	Yingkou, Liaoning	9382	142	46	Village
33	Oct. 8, 2018	Anshan, Liaoning	909	160	160	Farm

All Information of ASF outbreaks in China was retrieved through the World Organization for Animal Health Report and the Ministry of Agriculture and Rural Affairs of China

ASF is a hemorrhagic and often fatal disease of domestic pigs and European wild boar, and is notifiable to the World Organization for Animal Health (OIE). The disease is caused by African swine fever virus (ASFV), which is the sole member of the genus *Asfivirus* within the family *Asfaviridae*. ASFV is a tick-borne large DNA virus with complex transmission cycles among pigs, wild boar and soft ticks, and encodes more than 150 viral proteins with half of unknown functions. The virus shows high genetic and antigenic diversity. Up to now, 24 genotypes and 8 serogroups have been identified globally [4, 5].

## What has China done up to now?

The Ministry of Agriculture and Rural Affairs (MARA) of China has issued a number of policies and regulations for the prevention and control of ASF before and after ASF outbreaks in China (Table 2). Following confirmation of ASF outbreaks in China, standard measures have been implemented to control the disease, including culling all the pigs within 3 km of the epidemic area, harmlessly destroying all the infected pigs and animal disposals and contaminants. Till now, around 50 000 infected and affected pigs in the 33 ASF outbreaks in China have been culled (data from OIE), and the infected farms and contaminated materials were cleaned and disinfected. Following confirmation of the first ASF outbreak in China, the MARA immediately reported this event to the OIE on the same day and launched the ASF Contingency Plan and Emergency Response Level II. A series of regulations and actions have been taken by the MARA, including pig movement restriction inside the country, surveillance outside containment and/or protection zones, screening, quarantine, official destruction of pig products, official disposal of carcasses, by-products and wastes, stamping out, control of wildlife reservoirs, zoning, and disinfection, etc.

**Table 2:** Regulations issued by the Ministry of Agriculture and Rural Affairs of China for the prevention and control of African swine fever

No.	Dates	Regulations	Sources
1	Sept. 13, 2018	To stop using pig’s blood products as raw materials to produce pig feed. It is not allowed to feed pigs with untreated swills	MARA (Notice No. 64)
2	Sept. 11, 2018	To prohibit interprovincial transportation of pigs and related products from ASFV-affected provinces	MARA (No. 2018–33)
3	Aug. 10, 2018	To strengthen the supervision of pig transportation	MARA (No. 2018–38)
4	Aug. 3, 2018	To launch the ASF Contingency Plan and Emergency Response Level II	Information Office of MARA
5	May 7, 2018	To prohibit importing pigs, wild boar and related products directly or indirectly from Hungary.	MARA (Notice No. 35)
6	Feb. 14, 2018	About the prevention of ASF	MARA
7	Dec. 3, 2017	About the Implementation of Technical Specifications for Prevention and Control ASF (for trial implementation)	MOA
8	Oct. 20, 2017	About the implementation of ASF Emergency Plan	MOA
9	Apr. 14, 2017	Deployment to further strengthen risk prevention of ASF	MOA
10	Mar. 20, 2017	To strengthen the surveillance and epidemiological survey of ASF	MOA
11	Sept. 3, 2007	To prohibit to import pigs, wild boar and related products directly or indirectly from Armenia	MOA and AQSIQ (Notice No. 906)
12	Aug. 8, 2007	To prohibit to import pigs, wild boar and related products directly or indirectly from Georgia	MOA and AQSIQ (Notice No. 886)

*MARA* Ministry of Agriculture and Rural Affairs, formerly Ministry of Agriculture (MOA), *AQSIQ* Administration of Quality Supervision, Inspection and Quarantine

## Challenges for ASF control in China

China has the largest swine population in the world, with 688.61 million pigs fattened in 2017, accounting for approximately 48% of the world’s pork production (Source: Livestock and Poultry: World Markets and Trade, United States Department of Agriculture, April 2018). Meanwhile, small-scale and backyard farms with low biosecurity produce more than 60% pigs in China. A well-recognized high risk is swill-feeding currently practiced in many countries including China, which is a frequent route of ASFV introduction into ASF-free countries, e.g. Spain, The Netherlands, Belgium, Cuba, and Georgia [6]. A total of 20 ASF outbreaks occurred in farms mainly practicing swill-feeding in Anhui and Liaoning provinces of China. And illegal movement and slaughter of sick pigs before diagnosis pose another risk. The uneven distribution of the pig production in China makes long-distance pigs/pork transportation unavoidable, resulting in a higher risk of spreading ASF. Moreover, due to the endemic or epidemic situation of ASF in the Russian Federation and other countries, the disease has a high possibility to be reintroduced into China.

Another challenge is wild boar and soft ticks, which are natural hosts of ASFV and are widely distributed in China. We need to know if ASFV has taken root in wild boar and ticks after continuous outbreaks.

Though various vaccines against ASF are under development, none is commercially available. Difficulties in vaccine development need to be overcome, including safety concerns, poor cross-protection, and the lack of markers for sero-surveillance.

## Lessons learnt from other countries

China can learn a lot of lessons from other countries with a history of ASF. Take Spain as an example. ASF was introduced into Spain in 1967 and was not well controlled until 1985 when the European Union provided sufficient financial support to eradicate the disease. Spain established a network of mobile veterinary team and a reference laboratory for ASF surveillance and outbreak identification. Animal movement was under strict control and illegal pork transportation was forbidden. Reasonable compensation was provided for culling pigs [7].

Russia is another mirror for China. ASF has spread widely and become an endemic disease in Russia since the first introduction in 2007. The main reasons are: (1) illegal movement of infected pigs and pork products, swill-feeding, and improperly handling infected pigs; (2) circulation of ASFV in wild boar and anthropogenic factors; (3) lack of effective prevention and control measures and nationally funded eradication program; (4) absence of veterinary oversight for the large number of small holdings and backyard pig farms with low biosecurity [8, 9].

## Recommendations on ASF control in China

ASF is mainly transmitted by direct contact with infected pigs or ingestion of ASFV-contaminated pork products, etc. ASFV infection can also take place when the susceptible animals get physically in contact with ASFV. At present, no effective vaccines are commercially available and its control mainly relies on early detection and rapid eradication [10]. All the responsible stakeholders, including backyard farms, pig transporters, slaughter houses, veterinary services and authorities should join together to successfully implement the ASF control strategies. Effective measures must be adopted to control the disease.

To the Chinese government, with focus on coordination of surveillance and response activities:

- Establishing a multi-sectoral linkage mechanism, coordinating different departments to prevent and control ASF;
- Establishing multi-line barriers and defenses;
- Establishing a real-time monitoring, traceable pig/pork transportation system in line with biosecurity regulations; establishing inspection, quarantine and disinfection stations to control the movement of animal transportation vehicles from epidemic areas;
- Establishing a comprehensive surveillance and screen program and early test/reporting/warning and response system nationwide;
- Excluding small pig holders around the breeding units;
- Monitoring wild boar and ticks;
- Establishing a complete control system and strong technical support for disease prevention and control in epidemic, threatened and surrounding areas;
- Developing diagnostic assays for specific and early detection of suspected cases;
- Implementing a reasonable compensation policy;
- Owners of pig farms obligatorily to report any suspicious cases, ill or dead pigs to local veterinary authorities.

To pig owners, with focus on improving farm biosecurity measures:

- No introduction of pigs or semen from ASF-affected regions;
- Avoid carrying pork or related products from the outside to the farm;
- No addition of protein products of porcine origin in feed;
- No entry of visitors from ASF-epidemic regions;
- Clean and disinfect the trucks before and after pig transportation;
- Quarantine personnel for 1 to 3 days after traveling;
- Kill soft ticks, flies, and mosquitos.

## International collaborations on ASF

ASF is a formidable transboundary disease. Since the ever-increasing personnel exchange and globalization, ASF has a high risk to be introduced into any ASF-free countries, especially Asian countries. Therefore, the battle against ASF needs the international participation.

A safe and effective vaccine is a priority for the cost-effective control of ASF in countries with a large swine population. Joint efforts on vaccine development should be made among international laboratories, including identification of virulence- and protection-associated proteins, generation and comprehensive evaluation of gene-deleted vaccine candidates with complete as well as cross-genotype protection, serological markers and no side-effects, and development of immunological parameters and assays for vaccine evaluation, etc.

Other activities include transboundary and inter-regional surveillance of ASF, information sharing, and training of young scientists, especially epidemiologists.

## Conclusions

ASF is a highly devastating swine disease. Undoubtedly, the emergence and spread of ASF in China, the largest pork producer in the world, is an unprecedented disaster. To control ASF in China, where there are a large number of small holders and frequent pig/pork transportation, is a challenging and long-lasting battle that needs the joint participation and coordination of all stakeholders nationally and internationally.

## Additional file

Additional file 1: Multilingual abstracts in the five official working languages of the United Nations. (PDF 697 kb)

## Abbreviations

ASF: African swine fever
ASFV: African swine fever virus
MARA: Ministry of Agriculture and Rural Affairs
OIE: World Organization for Animal Health
