# Supplementary material for: African swine fever: an unprecedented disaster and challenge to China
Source: Infect Dis Poverty. 2018 Oct 26;7:111. doi: 10.1186/s40249-018-0495-3 (PMC6203974; doi:10.1186/s40249-018-0495-3)
Supplement: Supplementary file 1 — Multilingual abstracts in the five official working languages of the United Nations. (PDF 697 kb) [file 40249_2018_495_MOESM1_ESM.pdf]

Translation of the abstract into the five official working languages of the United Nations

تدابير التعرف على الثغرات البحثية في الأمراض المتنقلة وغيرها من أمراض الفقر المعدية في البيئات الحضرية: تقييم بروتوكول
حمى الخنازير الأفريقية: كارثة غير مسبوقة وتحدي تواجهه الصين

تاو وانغ ويوان سون وهوا جي تشيو

نبذة مختصرة

خلفية: حمى الخنازير الأفريقية (ASF)، الناجمة عن فيروس حمى الخنازيرالأفريقية، هو مرض نزفي قاتل غالبًا ويصيب الخنازير الأليفة والبرية ويجب الإبلاغ عنه للمنظمة العالمية لصحة الحيوان . وفي 3 آب /أغسطس 2018، أبلغت الصين عن أول حالة لتفشي حمى الخنازير الأفريقية (ASF) في مدينة شنيانغ، شمال شرق الصين. وفى 25 أيلول/سبتمبر، سُجلت 21 حالة تفشي في ثماني مقاطعات في الصين والتى تعتبر أكبر المقاطعات المنتجة والمستهلكة للحم الخنزير فى العالم .

الجزء الرئيسى: يلخص هذا التعليق الوضع الحالى لمرض حمى الخنازيرالأفريقية (ASF) في الصين ومختلف التدابير التي اتخذتها الصين ومازالت تتخذها، والتحديات والاقتراحات بشأن مكافحة مرض حمى الخنازيرالأفريقية بها، والدروس المستفادة من حالات حمى الخنازيرالأفريقية فى البلدان الأخرى، وإمكانية التعاون الدولي حول حمى الخنازير الأفريقية

الاستنتاجات: ستكون مهمة صعبة لم يسبق لها مثيل لمكافحة مرض حمى الخنازيرالأفريقية في الصين، الأمرالذي يتطلب مشاركة مشتركة وتنسيقًا بين الجهات المعنية والوكالات على مختلف المستويات.

Translated from English version into Arabic by Mais Salsa, proofread by Safaa Hassan, through

TRANSLATORS
WITHOUT BORDERS

非洲猪瘟：中国面临的空前灾难与挑战

王涛，孙元，仇华吉

摘要

引言：非洲猪瘟是由非洲猪瘟病毒感染家猪和野猪而引起的一种出血性、致死性传染病，世界动物卫生组织将其列为法定报告疫病名录。中国是全球最大的猪肉生产和消费国。2018 年 8 月 3 日，中国东北沈阳市爆发了首例非洲猪瘟疫情。截止 10 月 8 日，中国 8 个省份累计爆发了 33 起非洲猪瘟疫情。

主要内容：本文对中国当前的非洲猪瘟形势、已采取的防控措施、面临的挑战、国外防控经验、防控建议及国际合作前景进行了评述。

结论：中国的非洲猪瘟防控将是一场需要利益相关方共同参与和合作的艰苦持久战。

Translated from English version into Chinese by Tao Wang

La peste porcine africaine : un désastre sans précédent et un défi pour la Chine

Tao Wang, Sun Yuan, Hua-Ji Qiu

Extrait

Contexte: La peste porcine africaine (PPA), causée par le virus de la peste porcine africaine, est une maladie hémorragique souvent mortelle des porcs domestiques et des sangliers, qui est à notification obligatoire à l’Organisation mondiale de la santé animale. Le 3 août 2018, la Chine a signalé la première épidémie de peste porcine africaine à Shenyang, une ville du nord-est de la Chine. À compter du 25 septembre, un total de 21 foyers de PPA ont été enregistrés dans huit provinces en Chine, à savoir le plus grand producteur et consommateur de porc dans le monde.

Corps du texte: Ce commentaire résume la situation actuelle de la PPA en Chine, les diverses mesures appliquées par la Chine, les défis et les suggestions relatifs au contrôle de la PPA en Chine, les leçons apprises sur la PPA dans d’autres pays et les éventuelles collaborations internationales sur la PPA.

Conclusions: Ce sera une tâche difficile sans précédent de contrôler la PPA en Chine, nécessitant au passage la participation et la coordination conjointe des intervenants et des organismes à différents niveaux.

Translated from English version into French by Isabelle Mathis, proofread by Eve Anderson, through

TRANSLATORS
WITHOUT BORDERS

Африканская чума свиней: беспрецедентная катастрофа и проблема в Китае

Тао Ван, Юань Сунь, Хуа-Цзи Цю

Аннотация

Общие сведения: Африканская чума свиней (АЧС), возбудителем которой является вирус африканской чумы свиней, — геморрагическая, часто смертельная болезнь домашних свиней и диких кабанов, подлежащая обязательной регистрации во Всемирной организации по охране здоровья животных. 3 августа 2018 года Китай сообщил о первой вспышке АЧС в городе Шэньяне, расположенном в северо-восточной части страны. По состоянию на 25 сентября была зарегистрирована в общей сложности 21 вспышка АЧС в восьми провинциях Китая — крупнейшего производителя и потребителя свинины в мире.

Основная часть: В данном комментарии кратко излагается текущая ситуация с АЧС в Китае, различные меры, предпринятые государством, а также задачи и предложения по контролю АЧС на территории страны, кроме того, указан опыт борьбы с АЧС в других странах и возможность международного сотрудничества по АЧС.

Выводы: Контроль АЧС в Китае будет беспрецедентно трудной задачей, требующей совместной работы, а также координации деятельности заинтересованных сторон и организаций на разных уровнях.

Translated from English version into Russian by Polina Nikitina, proofread by Liudmila Tomanek, through

Peste porcina africana: un desastre sin precedentes y un desafío para China

Tao Wang, Sun Yuan, Hua Ji Qiu

Resumen

Antecedentes: La peste porcina africana (PPA), causada por el virus de la peste porcina africana, es una enfermedad hemorrágica a menudo mortal que afecta a cerdos y jabalíes, de notificación obligatoria a la Organización Mundial de Sanidad Animal. El 3 de agosto de 2018, China documentó el primer brote de PPA en Shenyang, una ciudad al noreste del país. A 25 de septiembre, un total de 21 brotes de PPA han sido registrados en ocho provincias de China, el mayor productor y consumidor de cerdo del mundo.

Cuerpo del texto: En este estudio se muestra la situación actual de la PPA en China, así como las diferentes medidas adoptadas por el país, los desafíos que supone la enfermedad y sugerencias para controlarla, consejos de otros países que la han sufrido y posibles acuerdos internacionales para combatirla.

Conclusiones: Controlar la PPA en China será una difícil tarea sin precedentes que requiere una participación y coordinación conjunta de los accionistas y los organismos a varios niveles.

Translated from English version into Spanish by David López Canelada, proofread by Tanya Fernández Escudero, through
